# Reproductive Tactics of Sexes and Fitness in the Dragonfly, *Diastatops obscura*

**DOI:** 10.1673/031.007.2401

**Published:** 2007-04-16

**Authors:** Jorge Bañuelos Irusta, Arrilton Araújo

**Affiliations:** Sector of Psychobiology, Department of Physiology, Federal University of Rio Grande do Norte (UFRN), Caixa Postal 1511 - Campus Universitário, 59072-970, Natal-RN, Brazil

**Keywords:** sexual selection, female choice, dragonfly, Pitimbu River

## Abstract

The sexual selection strategies of territorial Odonata that do not present courtship behavior is still not completely understood, especially the role of the females. *Diastatops obscura* Fabricius (Odonata: Libellulidae) females participate in mate selection in a passive manner, allowing copulation with the first male that captures them and afterwards choosing whether to oviposit or not. This study introduces the idea of female passive choice as an adaptative tactic in intersexual selection. Also discussed is the adaptative value of this tactic and its flexibility according to environmental conditions and reproductive strategies adopted by the males. A natural population of *Diastatops obscura* was observed in the Pitimbu River of northeast Brazil. Focal continuous and ad libitum techniques were used to record attempted copulation, copulation, and oviposition behavior, in addition to registering male territoriality. An estimate of individual reproductive success (IRS) was obtained by recording 187 reproductive events. Territorial males, mainly occupying areas near the river margin, achieved greater copulation and oviposition success (IRS = 0.371) than did satellite males (IRS = 0.028). Females that copulated with territorial males experienced, for the most part, only one copulation and oviposition event, while those that copulated with satellite males fled or performed a second copulation with a territorial male. Thus, the best tactic adopted by the *D. obscura* males was to occupy a territory providing the greatest access to females, while the females used passive choice for fitness optimization.

## Introduction

In animals that do not provide parental care, mates do not actively contribute to the survival of descendants. The main benefit sought after in sexual selection is indirect access to the genetic load and its transmission to descendants, while lost opportunities (Wilcox 1984) and possible injuries suffered in the successive rejections of unfit males (Blanckenhorn et al. 2000; Cartar 1992) are the main costs associated with this choice. If the operational sex ratio is male biased, female choice would minimize the first of these costs by having a large number of males available. This would reduce mate selection waiting time, but the risk of injury could rise as a result of the increased pressure of males attempting to mate, which could cause females to relax their selection criteria, culminating in convenience polyandry (Rowe 1992).

When there is territoriality females must rely on clues that indicate male quality, such as territory characteristics, secondary sexual features or courtship behavior, all of which will influence the mate selection strategies adopted (Gontard-Danek and Möller 1999; Luttbeg 2004). In territorial dragonflies, such as *Diastatops obscura* Fabricius (Odonata: Libellulidae), without courtship behavior, female sexual selection strategies are not yet fully understood. Among the direct benefits that the female derives from finding a dominant male, are oviposition in a high quality territory and postcopulatory guarding by the male during oviposition (Corbet 1999; Sherman 1983; Tsubaki et al. 1994). In regard to the first benefit, Tsubaki and Ono (1987) suggest that territory, rather than male characteristics, defines mate selection. Koenig (1991) showed that females showed a preference for males occupying high quality territories over those from resource-poor territories or satellite males, but that their interest in reproductive sites seemed to be based more on minimizing the time spent in these areas than on optimizing sexual selection. On the other hand, Fincke (1988) discounted the importance of female participation and suggested that differences in male reproductive success are a result of intramale competition. We suggest that there is greater indirect female participation in mate selection among territorial libellulids, indicating passive sexual selection linked to territorial competition among males.

The *D. obscura* mating system was recently described by Irusta and Araújo (2006). The males of this species arrive at the reproductive sites before the females and compete with other conspecific males in an attempt to occupy, defend or conquer a territory. In these interactions, hierarchies and reproductive tactics are established, where dominant territorial, secondary territorial and satellite males are differentiated. When sexually mature females arrive in these areas to mate and lay their eggs, they are captured by either territorial or satellite males as they approach, without their offering any apparent resistance. They are generally captured by the first male in their flight path that then directs her in tandem into his territory where a brief copulation (3–6 seconds) and oviposition occurs under non-contact guarding.

Thus, it is expected that males defending territories near the river margins have a better chance of obtaining females. These highly disputed territories are occupied by males capable of conquering and defending territory and, thereby, offering good reproductive perspectives to their mates. As shown here, these territorial males are also a resource sought by females in the reproductive areas. The selection procedure is performed indirectly when females allow themselves to be captured by one of the territorial males along the river margin and form a tandem. We thereby introduce the concept of female passive choice as an adaptative strategy of intersexual selection in Odonata females.

Three predictions are suggested to explain the preference of males for territories that offer greater access to females and the preference of females for these more successful territorial males:

First prediction: *D. obscura* males will compete to occupy territories near the river margin that provide greater access to females. Since there are no courtship or secondary sexual features indicating female dominance, the males defend a territory in the river margin, the area most sought-after by other males. Mid-river territories are also convenient for egg and larval development, as demonstrated by larvae collecting activities, but only a few females venture there because of the males present at the river margin.

Second prediction: Males that occupy territories at the river margin will have better reproductive success. The preference for margins must be linked to better fitness in the males that conquer these territories. This implies a female preference for this type of territorial male and a higher oviposition rate for females fertilized by them.

**Figure 1.**
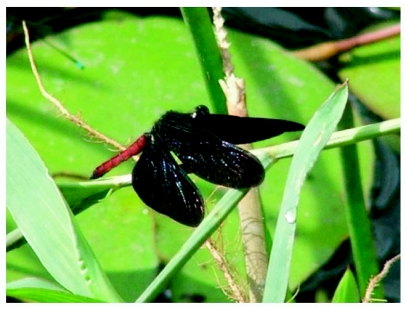
Typical display of *D. obscura* male in territorial behaviour. Photo: J.B. Irusta.

Third prediction: Females will more readily accept a second copulation when the first has occurred with a satellite male. When there is no courtship, female libellulids do not seem to be able to discern *a priori* among the qualities of different mates. This occurs only after tandem formation when they are taken to a territory of greater or lesser quality to complete copulation (Convey 1989; McVey 1988). The prediction is that females that copulate with satellite males will more often accept second copulations, while females that mate with territorial males will avoid subsequent copulations, and opt more frequently for immediate oviposition.

## Materials and Methods

*D. obscura* is a small neotropical Libellulid with wing characteristics that partially determine its behavior. Its short, wide wings make impossible the rapid and powerful flight that is typical of other members of the Odonata order. Males have bright dark-blue wings that seem to perform thermoregulation and intraspecific visual communication functions (Irusta and Araújo 2006; Wildermuth 1994, for *Diastatops intensa*), and a bright-red abdomen (Figure 1). Females have darker wings and a chesnut colored abdomen. Contrary to other Palpopleurinae, there is no male courtship of the female.

The observations were made in natural populations of the Pitimbu River (S: 05°56′04,6″, W: 35°10′50,0″) (Parnamirim-RN, Northeast Brazil) in four study periods: September, 2002 (4 days), April–May, 2003 (15 days), July, 2003 (5 days) and February, 2004 (8 days), totaling 92 hours of observation. The Pitimbu River is a small permanent waterway (7 to 9 meters wide at the study site), with a quadrangular section (0.8–1.0 m. deep from margin to mid-river) and a slow stream; there is abundant natural vegetation, both aquatic (along the entire river-width) and marginal (further details in Irusta and Araújo 2006).

At the study station two perpendicular axes were defined that allowed the determination of the position of each individual or territory at all times. On the axis parallel to the river margin, 7 small flags were placed at 3 meter intervals thus delineating the 7 continuous stretches where most of the observations were made. The perpendicular axis measured the distance to the margin.

To better identify and observe the individuals, 135 males were captured. The wings were marked with a permanent marking pen, and then released immediately at the capture site. Approximately one-third of the males present in the area were captured. The marking of individuals was performed on different days and/or times from those selected for behavioral observation, thus avoiding influencing the natural behavior of the dragonflies by either manipulating or disturbing them. In the pilot study, females arriving in the reproductive area were also captured, however, this practice was discontinued because marked females fled and did not return to the research area, making it impossible to observe any mating behavior. Females were also marked away from the reproductive site but since none were observed approaching the study area, we decided to proceed without any manipulation whatsoever of the females. In spite of not being marked, the low density of females during rendezvous with males enabled, in most cases, a differentiated observation of individual female reproductive behavior. A total of 161 females were observed entering the study area, resulting in 187 reproductive events (attempts at copulation, copulation and ovipositions).

The *ad libitum* (unconstrained) observation method was used during the first interactions between the males, at which time they competed to establish their daily territories. Continuous focal observation was used to monitor females in the reproductive areas, recording, whenever possible, breeding females' activities until their departure. The duration of observation was determined by the reproductive events themselves and by the dragonfly's remaining within the field of vision of the observer. In most of the observations, low female density and short-duration stay at the reproductive area enabled their individualized monitoring. When this was not possible, the data were disregarded.

Aspects related to the territorial and reproductive activities of individuals were quantified in the stretch of river studied: level of occupation of the rendezvous (*sensu* Corbet 1999), density of the territorial males, interactions between individuals and activities linked to reproductive behavior such as copulations and attempts at copulation, postcopulatory oviposition, second copulations and attempts at second copulation, oviposition after second copulation and third copulations. Second copulations are defined as those occurring shortly after the first one, but with a different male. Attempts at copulation and second copulation refer to contacts in which a male succeeds in holding a female for a brief period but does not manage to maintain the tandem position or maintains it for less than two seconds, making an effective copulation impossible. Nevertheless, an attempt at second copulation always implies the interruption of the female's previous situation, mainly the end of the male's guarding behavior and oviposition. Oviposition, in this paper, means oviposition immediately after copulation; any other egg-laying without immediate previous copulation was disregarded. All of these mating behavior aspects were associated to each of the three different tactics used by the males present in the reproductive areas: territorial males at the river margin, territorial males at mid-river and satellite males in riverbank vegetation near the marginal territories. Margin, in this paper, corresponds to the lateral strip of river next to the riverbank, permanently under water and with abundant aquatic and semi-aquatic vegetation. Satellite males were considered to be the non-territorial males that remained near, but outside the aquatic territorial area, on the side traversed by incoming mature females.

The study was designed to demonstrate three preestablished predictions described above. To verify the first one (male competition for margin territory), the occupation densities of males in territories situated at the margin and in the central areas of the river and their variation over the course of the day was compared. The times when territories were occupied and abandoned were also recorded.

To demonstrate the second prediction (related to the reproductive advantages associated to males who succeed in defending territories at the margin), individual reproductive success (IRS) was observed for each of the three tactics adopted by the males.

IRS = [number of postcopulatory ovipositions × fertilization rates (1.00) + number of copulations without immediate oviposition × fertilization rate (0.05) + number of ovipositions after second copulation × fertilization rate (0.95)] / number of males using this tactic.

**Figure 2.**
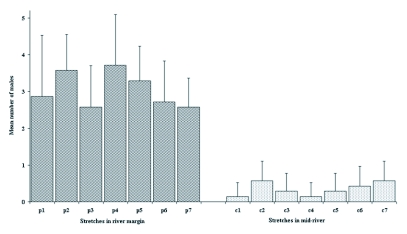
Male occupation (mean ± SD) per stretch in river margin and mid-river. p1-p7: stretches in river margin; c1-c7: stretches in mid-river.

We estimate that single copulations followed by immediate oviposition fertilize all the eggs (100% fertilization rate) and that later copulations followed by oviposition fertilize 95% of the eggs. The remaining 5% of the eggs are likely fertilized by males responsible for earlier copulations. These percentages are based on the data of previous studies dealing with libellulids (Corbet 1999; McVey and Smittle 1984; Siva-Jothy and Tsubaki 1994; Córdoba-Aguilar et al. 2003). These values demonstrate the importance of sperm precedence among libellulids.

**Figure 3.**
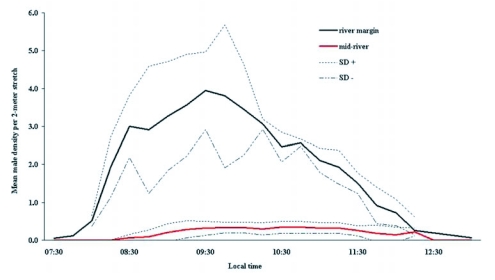
Time variation of male density at river margin and mid-river.

In the third prediction (when females mating with satellite males accept more readily a second copulation with another male, but do not subsequently copulate with territorial males) the percentage of second and third copulations of the females were compared, according to each of the tactics adopted by the males responsible for the first copulation.

Prior to analyzing the reproductive data, the seven continuous river sections into which the study area had been divided were compared. This was done to determine if there were environmental differences among them that could produce different occupation responses in the territorial males. Aspects such as the composition and relative abundance of aquatic macrophytes (estimated in %), lamina cover (in %), visibility level at the margin (in %), depth (m) and subsurface current velocity 1m. from the riverbank and at mid-river (m/s) were analyzed for each section at different times.

**Figure 4.**
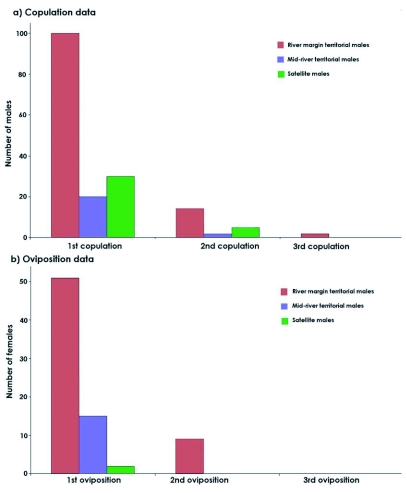
Comparative results of male reproductive tactics: a) Copulation data; b) Ovoposition data. Note the different scale.

The degree of male occupation of the marginal and central areas of the river was compared through Student's t-Test for independent variables and bilateral verification. The reproductive success of each of the male tactics was compared using bilateral chi-square test (χ^2^). The significance level used was 5%.

**Figure 5.**
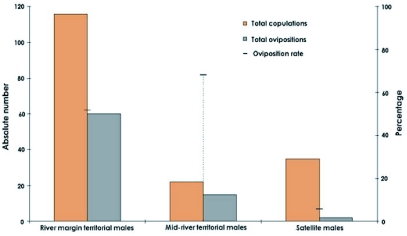
Reproductive success of male tactics.

## Results

The sections of both the marginal (ANOVA: F=1.16, df=48, p>0.05) and central areas (F=1.00, df=48, p>0.05) of the studied river section were compared first. No significant differences were found among their components (Figure 2), which agrees with the homogeneity encountered for the environmental parameters analyzed. This allowed the treatment of the margin and the mid-river as two intrinsically homogeneous areas. The degree of occupation of the marginal and central areas was compared and a significant preference for the former was observed in all the sections studied (t= 12.68, df=12, p<0.05) (Figure 2), thus confirming the first hypothesis.

In the variation analysis of mean male density in the marginal and central territories, according to the time of day of occupation, the difference between the two groups was verified. The fact that marginal area occupation occurred earlier than it did in the central areas, indicates that the first males to arrive there try to occupy these territories (Fig. 3). In accordance with the second prediction, male sexual activity in marginal territories is much more prevalent than that of males who adopt other tactics. Significantly higher values for the total number of copulations (*χ*^2^ = 89.98; df = 2; p<0.0001; Figure 4a) and for ovipositions (*χ*^2^ = 72.18; df = 2, p<0.0001; Figure 4b) were recorded. When compared to territorial males in the central area and satellites, significant differences were found in the number of ovipositions (*χ*^2^ = 9.94; df = 1; p = 0.0016), but not in the number of copulations (*χ*^2^ = 2.96; df = 1; p = 0.085). The density of territorial males at mid-river does not reach high levels (0.13±1.10 males/linear m., mean ± SD), which leads to 68.0% of the copulations in this area being followed by female guarding and oviposition.

**Table 1.**
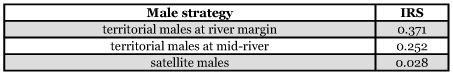
Estimate of individual reproductive success (IRS) for each tactic adopted by the males.

The number of successful copulations by satellite males, especially in times of high density, surpassed that of territorial males at mid-river. However, this apparent advantage was dispelled when the oviposition rate of recently fertilized females was determined, which is more closely related to the reproductive success associated with each of the tactics adopted by the males. Only two brief ovipositions were observed after copulation with satellite males (which represents 5.7% of the copulations), while a much higher oviposition rate was found for copulations with territorial males, both at the margin (52.0%) and at mid-river (68.0%) (Figure 5). Based on these results, the individual reproductive success (IRS) associated with each of the three tactics adopted by the males in the study area was estimated (Table 1).

The results of female receptivity to a second copulation support the third hypothesis. Thus, the majority of copulations with satellite males were followed by second copulations (or copulation attempts) of the female by other males (56.7%) or by spontaneous flight from the area when the female was released by the satellite male (36.7%) after copulation; both situations led to failed oviposition. Females that copulated with territorial males at the margin or at mid-river only copulated a second time (or suffered copulation attempts) in 23.0% and 20.0% of the cases, respectively.

It is worth pointing out the different attitude of females after copulating with a satellite male or a territorial male. In the first case, they were poorly guarded by the satellite males, who abandoned them as soon as they encountered territorial males at the river margin in order to avoid being attacked by these usually stronger and more aggressive males. The females appeared to allow a second copulation in the areas defended by territorial males at the river margin. Contrarily, females that initially copulated with territorial males had second copulations at a much reduced rate (9.3%), especially in low male density areas. Their attitude, marked by fleeing and hiding in vegetation in an attempt to avoid further copulations, revealed an obvious resistance to new encounters.

## Discussion

The adaptability of a determinate behavior can be analyzed by comparing estimates of the reproductive success of the different strategies adopted. In male dragonflies this success is usually measured directly according to the order of copulation (sperm precedence), the number of copulations and the number of eggs deposited immediately after copulation or indirectly by the duration of copulation, while for the females the genetic quality of the male and the egg-producing capacity of the female herself is what matters most (Corbet 1999).

The adaptative value of the apparent passivity in the sexual selection of the female *Diastatops obscura* must be understood in relation to the reproductive strategies adopted by males. Of special importance are the density of territorial males and the proportion of satellite males near the reproductive areas. Thus, in low male densities, with few or no satellites, females show little resistance to the first copulation, clearly avoiding subsequent mating (Irusta and Araújo 2006). However, high male densities at the rendezvous favor an increased number of satellites (as described for *Libellula quadrimaculata*, Convey 1989) that manage to copulate with a significant proportion of the females in the reproductive area. In these cases female passive choice tactics, which involves mating with the first male encountered, does not appear advantageous since she may not be captured by a territorial male.

The copulations with satellite males are completed away from the oviposition sites, thus avoiding interactions with territorial males, which seem to indicate the inferior condition of the male (Convey 1989; McVey 1988). This causes the female to change her usual behavior (immediate non-contact-guarding oviposition), in which she escapes and/or seeks a second copulation in an area defended by territorial males. This behavior was suggested for other female libelullids such as: *Leucorrhinia intacta* (Wolf et al. 1989), *Pachydiplax longipennis* (Sherman 1983), *Libellula luctuosa* (Moore 1989) and *Plathehis lydia* (Koenig 1991). Thus, not only do male libellulids exhibit a previously known flexibility in reproductive strategies that vary according to environmental and population conditions, but the females also modify their mate selection strategy according to the density and status of the males.

Territorial males of central river areas maintained a mean IRS rate similar to that of territorial males at the river margin, despite the limited number of females that reach these areas (Table 1). This can be explained by the lower male density at these sites, favoring a high percentage of postcopulatory ovipositions in these territories. Higher densities would decrease this index rapidly, removing adaptativity from this tactic.

Satellite males exhibited a very low reproductive success rate, despite the reasonable number of copulations, since the females usually escaped or sought subsequent mating, significantly limiting the satellite males' egg fertilization rate. Furthermore, the few females guarded by satellite males that managed oviposition, achieved it only for a few seconds, since they were quickly approached by other males who easily dominated the weak mating guard of the satellites. This further minimized the reproductive success of the satellite males, given that the duration of female oviposition is a parameter directly linked to the reproductive success of the males that fertilize them (Corbet 1999; Sherman 1983).

Sperm competition in Odonata, caused by functional and morphologic modifications of the aedeagus, ensures a high fertility rate in recopulations followed by oviposition (around 95% in the libellulids studied: see McVey and Smittle 1984; Michels 1989; Siva-Jothy and Tsubaki 1994). It is likely that for *D. obscura*, the sperm precedence values are similar to those indicated for other species of the family, since their aedeagus has a fourth segment with wide, flat, spiny lobes on the inner surface (Pujol-Luz, Pujol-Luz JR. Department of Zoology, Institute of Biological Sciences, University of Brasilia. Instituto de Ciências Biológicas, Asa Norte. 70910900 - Brasilia, DF, Brazil, personal communication), which is apparently adapted for sperm removal. Sperm precedence seems correlated with the inability of males to differentiate between female virgins and non-virgins (see Uhía and Cordero Rivera 2005, for damselflies). This is not conducive to male satellite success and further justifies the female strategy of allowing copulation with another higher-status mate when the initial mating was with a satellite male.

The differences encountered in estimating reproductive success associated with male strategies explain the female reaction when captured by a satellite male, where she abandons her passive attitude in order to search for another copulation in areas usually defended by territorial males. This behavioral change definitively supports the hypothesis of female selection, demonstrating that females do in fact participate in sexual selection by seeking a quality male to fertilize their eggs. This is done in an indirect and normally passive manner, and favors males victorious in territorial competition in reproductive areas.

## **Abbreviations**

IRS: individual reproductive success
